# Association of persistent pain with the incidence of chronic conditions following a disabling work-related injury

**DOI:** 10.5271/sjweh.4096

**Published:** 2023-07-01

**Authors:** Kathleen G Dobson, Cameron A Mustard, Nancy Carnide, Andrea D Furlan, Peter M Smith

**Affiliations:** 1 Institute for Work & Health, Toronto, Ontario, Canada.; 2 Dalla Lana School of Public Health, University of Toronto, Toronto, Ontario, Canada.; 3 Toronto Rehabilitation Institute, UHN, Toronto, Ontario, Canada.; 4 Division of Physical Medicine and Rehabilitation, Department of Medicine, University of Toronto C. Toronto, Canada.

**Keywords:** arthritis, back problem, diabetes, migraine, mood disorder, prevalence, worker compensation, working-age adult

## Abstract

**Objectives:**

In a cohort of workers disabled by a work-related injury or illness, this study aimed to: (i) compare pre-injury prevalence estimates for common chronic conditions to chronic condition prevalence in a representative sample of working adults; (ii) calculate the incidence of chronic conditions post-injury; and (iii) estimate the association between persistent pain symptoms and the incidence of common chronic conditions.

**Methods:**

Eighteen months post-injury, 1832 workers disabled by a work-related injury or illness in Ontario, Canada, completed an interviewer-administered survey. Participants reported pre- and post-injury prevalence of seven physician-diagnosed chronic conditions, and demographic, employment, and health characteristics. Pre-injury prevalence estimates were compared to estimates from a representative sample of workers. Multivariable logistic regression was used to examine the association of persistent pain with post-injury chronic condition incidence.

**Results:**

Age-standardized pre-injury prevalence rates for diabetes, hypertension, arthritis, and back problems were similar to prevalence rates observed among working adults in Ontario, while prevalence rates for mood disorder, asthma and migraine were moderately elevated. Post-injury prevalence rates of mood disorder, migraine, hypertension, arthritis, and back problems were elevated substantially in this cohort. High persistent pain symptoms were strongly associated with the 18-month incidence of these conditions.

**Conclusions:**

The incidence of five chronic conditions over an 18-month follow-up period post injury was substantial. Persistent pain at 18 months was associated with this elevated incidence, with population attributable fraction estimates suggesting that 37–39% of incident conditions may be attributed to exposure to high levels of persistent pain.

One of every six injuries requiring medical attention among working-age adults in North America are caused by work exposures (1). Injuries and illnesses caused by, or associated with, work exposures have substantial societal economic impact in both developed (2) and developing countries (3). Globally, they account for more than 3% of all disability-adjusted life years in the world’s population (4). The approximately 35% of work-related injuries and illnesses that result in periods of disability and work absence are responsible for the majority of this economic burden (2).

Work-related injuries also result in consequences to workers’ long-term health, including deficits in physical health (5), mental health (6), and the onset of chronic conditions (7). The relationship between traumatic injury and chronic conditions has largely been examined from two research perspectives. From a first perspective, injury epidemiology in workplace and non-workplace settings has assessed the degree to which the risk of traumatic injury is increased among adults with prevalent chronic condition disorders or sensory impairments (8–11). For example, a study based on national cross-sectional survey data found that working-aged Canadians with arthritis or back problems were at increased risk of experiencing a work-related injury (8). Underlying this perspective is that there may be pre-existing factors, which predispose a worker to both a chronic condition and a work-related injury. From a second perspective, clinical epidemiology studies have documented the degree to which recovery and rehabilitation following traumatic injury are impaired among patients with pre-existing chronic conditions (12, 13). This literature suggests that injured workers who were previously diagnosed with depression, diabetes, coronary heart disease, or osteoarthritis experience longer durations of wage replacement benefits (13, 14).

Less attention has been given to a third perspective: the extent to which traumatic injury may predispose or accelerate the incidence of chronic conditions among working-aged adults. In one study of young US veterans, comparing 10 000 combat-injured personnel to a matched cohort with no combat injury, there was a moderately elevated incidence of hypertension and diabetes among the combat-injured over a median follow-up period of approximately eight years (15, 16). Other studies have assessed the prevalence of chronic conditions following injury. For example, among a longitudinal cohort of US construction workers, the prevalence of hypertension, arthritis, diabetes, back problems, and depression assessed at 40 years of age was elevated among workers who experienced a traumatic work-related injury in the preceding 10 years compared to non-injured workers (17). An elevated prevalence of mood disorders following work-related injury has also been found in both short and long follow-up durations in longitudinal cohorts (6, 18–21).

Among the consequences of traumatic injury is a substantial incidence of persistent or chronic pain (22). In a recent analysis of the cohort of workers used in this current study, 24.9% reported severe pain intensity with substantial functional impairment in an interview 18-months post-injury (23). The prevalence of severe persistent pain in this cohort is approximately six times higher than in Canadian and US adult populations, where 4% of adults experience persistent pain with substantial functional impairment (24, 25). There is a growing recognition of the etiologic contribution of traumatic injury to the population burden of chronic pain, leading to proposals to revise the classification of chronic pain in ICD-11 to include ‘chronic post-surgical and posttraumatic pain’ as one of seven etiologic categories (26). One specific syndrome associated with chronic pain, posttraumatic arthritis, is estimated to account for 10–12% of all cases of symptomatic osteoarthritis among adults, where persons who sustain a traumatic knee injury are four times more likely to develop osteoarthritis than those without a history of knee injury (27).

This study pools two samples of workers experiencing a disabling work-related injury or illness to estimate the association between persistent pain 18 months post-injury and the incidence of physician-diagnosed chronic conditions. The study has three objectives: to (i) compare pre-injury prevalence of common chronic conditions to chronic condition prevalence in a representative sample of working adults, (ii) calculate the incidence of chronic conditions post-injury, and (iii) estimate the association between persistent pain symptoms and the incidence of common chronic conditions in the 18-month period post-injury. The study design oversampled disability benefit recipients with longer duration disability episodes and incorporated measures obtained at baseline from administrative records of workers’ compensation benefits with measures obtained from interviews with cohort members 18 months following the incidence of disabling injury or illness.

We hypothesized that, once taking into account the length of claim duration, the prevalence of chronic conditions among cohort members before the disabling injury would be equivalent to prevalence rates observed in a representative sample of working adults in this setting. We also hypothesized that high persistent pain symptoms would be associated with a greater probability of incident chronic conditions (28). The incidence of chronic conditions in an 18-month follow-up period among cohort members may potentially be related to two contrasting mechanisms. First, the therapeutic management of a work-related injury may include diagnostic testing that results in a diagnosis of previously unrecognized disorders. Second, an increase in the frequency of diagnosed chronic conditions may be due to disorders, or lifestyle changes increasing the risk of disorders, that are secondary to the work-related injury. For example, traumatic injury to the musculoskeletal system may result in an increase in the diagnosis of posttraumatic arthritis or injuries resulting in persistent pain may result in an increase in the diagnosis of migraine or mood disorder.

## Methods

### Setting

Approximately 70% of the seven million labor force participants in Ontario, Canada are employed by organizations that have a mandatory obligation to obtain work disability insurance coverage from the publicly administered, single-payer workers’ compensation insurance authority: the Workplace Safety & Insurance Board (WSIB). The WSIB administers benefits to entitled workers, covering medical care services and the provision of wage replacement benefits for workers whose recovery from a work-related injury or illness requires absence from work. In 2018, the WSIB administered benefits for approximately 200 000 compensation claims, of which 67 000 were claims resulting in one or more days absent from work.

### Sample

The Ontario Life After Work Injury Study (OLAWIS) pools information from two cohorts of workers in Ontario, Canada, who were disabled by a physical work injury or illness (herein referred to simply as injury) and received wage replacement benefits. Both cohorts were comprised of Ontario workers ≥18 years old, who were employed by an insured employer, able to conduct an interview in English or French, and had experienced a physical work-related injury or occupational disease that resulted in a WSIB accepted compensation claim for wage replacement benefits. To obtain sufficient representation of more serious and complex disability episodes, participants with longer wage replacement durations of three months or greater were oversampled. Workers with wage replacement benefit durations of one day to three months, representing 85% of all accepted claimants in this setting, comprised 41% of the combined sample.

Participant recruitment for the first cohort, OLAWIS1, occurred between June 2019 and March 2020, among workers disabled by a work-related injury in the period from January to October 2018. From randomly sampled monthly quotas of lost-time claimants meeting eligibility criteria, 2816 claimants were contacted, of which 1132 completed an interviewer-administered telephone interview (40.2% of eligible claimants). Among participants, 358 (31.6%) were in the short-duration claim sample and 774 (68.3%) were in the long-duration claim sample. More details on the OLAWIS1 study cohort may be found elsewhere (29).

Participant recruitment for the second cohort, OLAWIS2, occurred over the period September to November 2021. From a census of all claimants disabled by a work-related injury or illness in January or February 2020, 2309 randomly sampled claimants were contacted, of which 700 claimants (30.3% of eligible claimants) completed interviews. Among OLAWIS2 participants, 395 (56.4%) were in the short-duration claim sample and 305 (43.6%) were in the long-duration claim sample.

Compared to potential participants who consented to the study but did not complete the survey, no differences were present in terms of age, gender, geographic location, industry, and employer size. However, duration of benefits was slightly longer among participants versus non- participants. Of those participants who completed the survey, a greater proportion of participants with medium (3–12 month) and long-term (12–18 months) claims agreed to link their survey response with their administrative claim data.

The present study included all cohort members of the OLAWIS1 (N=1132) and OLAWIS2 cohorts (N=700), 1832 participants. The Health Sciences Research Ethics Board at the University of Toronto granted Research Ethics Board approval (Protocols 37525 and 41560).

### Data sources

*OLAWIS1 and OLAWIS2.* Primary outcome measures and potential predictors of the primary outcomes were drawn from WSIB administrative records and an interviewer-administered telephone questionnaire 18 months after the original injury occurred. Information from an 18-month follow-up of administrative records of work disability insurance benefits, providing a measure of the nature of injury, was linked with the information obtained from the interviewer-administered questionnaire – 94% and 84% of OLAWIS1 and OLAWIS2 participants, respectively, consented to this linkage. To maximize statistical power, the two cohorts have been pooled.

*Canadian Community Health Survey.* The Canadian Community Health Survey (CCHS) is an ongoing series of cross-sectional health interview surveys administered approximately every two years by Statistics Canada, the Canadian federal statistics agency (30). Using a multi-staged, stratified sampling frame, the CCHS target population consists of household residents aged ≥12 years old who are living in private dwellings in all Canadian provinces and territories. The survey sample design and core content have remained largely unchanged during the series of surveys starting in 2001. In this study, the CCHS analysis sample was drawn from the 2015–2016 survey cycle, restricted to 13 700 Ontario adults 20–64 who reported working ≥15 hours in the week of the survey.

### Study measures

*Chronic conditions.* The measure of self-reported prevalence of chronic conditions applied to the OLAWIS cohorts replicated the questionnaire wording in the CCHS, asking respondents: “Now I’d like to ask about certain long-term health conditions which you may have. We are interested in long-term conditions which are expected to last or have already lasted ≥6 months and that have been diagnosed by a health professional.” The analysis reported in this study focused on seven conditions with substantial prevalence among working-aged adults: diabetes, mood disorder, asthma, migraine, hypertension, arthritis, and back problems. For each chronic condition, response options in OLAWIS included: “No”, “Yes, condition present before the work injury”, or “Yes, condition present after the work injury”. In the present study, we focus on both prevalent conditions (ie, those that occurred before the work-related injury), and incident conditions (those that occurred after the work-related injury).

The validity of physician-diagnosed chronic conditions obtained from survey respondent self-report has been extensively evaluated in comparison to reference standards based on clinical diagnostic evaluation (31) and diagnostic information reported in health care utilization records (31–34). While these studies typically document a degree of discordance between measurement methods, there is, in general, a consensus that self-reported prevalence information on chronic condition prevalence is valid for population-based surveillance of chronic conditions (32–34).

*Persistent pain interference.* OLAWIS participants were asked about current levels of pain interference with normal activities (“During the past 4 weeks, how much did pain interfere with your normal work (including both work outside the home and housework)?”), to which participants could respond: not at all, a little bit, moderately, quite a bit, or extremely (35).

*Demographic, work, health, and injury variables.* The factors explored as covariates of the relationship between persistent pain and incident chronic conditions in this study include participant age, sex, highest level of education, employment status 18 months post-injury (permanent, temporary, not working), and the industry in which one was working in at the time of the injury. In the survey, participants were also asked about their healthcare experience, including if they were currently receiving healthcare for their injury. Nature of the work-related injury was obtained from administrative records, classified in five categories: superficial or open wound; sprain, strain or dislocation; fracture; internal injury (including concussion); or other. Those not agreeing to the use of their administrative records were coded as a “missing” category for analyses. We also included cohort (OLAWIS 1 or 2), as well as length of benefit duration (≤3 months) as covariates.

### Analysis

Cohort-specific pre-injury and post-injury prevalence rates for seven chronic conditions were calculated, age-standardized to the age distribution of Ontario workers in the CCHS. Applying the Fay method, 95% confidence intervals (CI) were computed (36).

The distribution of demographic, work, health, and injury measures were estimated for incident cases of asthma and for incident cases of three groups of disorders: arthritis and/or back problems, hypertension and/or diabetes, and mood and/or migraine disorders. Disorders were grouped together due to their common etiology and co-occurrence (37–39). As the focus of analysis were incident cases of chronic conditions diagnosed after the work injury, participants who reported having the condition prior to the work injury were excluded.

For the three incident chronic condition groups, logistic regression was used to calculate crude odds ratios of the association with persistent pain, as well as adjusted odds ratios from models including all covariates [crude odds ratios (OR) are available in the supplemental file]. Due to the small number of cases (N=18), incident asthma was excluded from these analyses. All analyses were weighted by the probability of selection to account for the oversampling of longer duration disability episodes. Sample weights were calculated as the inverse of the sampling fraction, which is estimated as the number of survey participants in a sample group divided by the number of eligible claimants in the sample frame. Sample weights were normalized. Analyses were completed in R software (version 4.2.0).

Regression analyses were conducted using a complete case framework. As such, the regression for incident arthritis or back pain included 1695/1720 eligible participants (observations missing from analysis, N= 25; incident cases missing from analysis due to missing covariate information, N=3). The logistic regression for incident hypertension or diabetes included 1728/1753 eligible participants (observations missing from analysis, N=25; incident cases missing from analysis due to missing covariate information, N=3), and the regression for incident migraine or mood disorder included 1739/1763 eligible individuals (observations missing from analysis, N=24; incident cases missing from analysis due to missing covariate information, N=4).

Population attributable fractions, estimating the fraction of incident chronic conditions attributable to exposure to severe persistent pain, were calculated from adjusted OR obtained from multivariable regressions (40). The formula applied was the product of the percent of incident chronic condition cases exposed to persistent pain multiplied by (1 – 1 /OR), where the OR was obtained from multivariable regressions.

## Results

Table 1 reports the pre- and post-injury prevalence rates for seven common chronic conditions, stratified by the two OLAWIS cohorts and compared to the representative sample of working adults in Ontario. In both cohorts, the age-standardized pre-injury prevalence rates for diabetes, hypertension, arthritis, and back problems were similar to prevalence rates observed among working adults in Ontario. Conversely, in both OLAWIS cohorts, the age-standardized pre-injury prevalence rates for mood disorder, asthma and migraine were moderately elevated compared to Ontario working adults.

**Table 1:** Prevalence of seven chronic conditions pre-injury and 18 months post-injury: self-reported physician diagnosis Ontario Life After Work Injury Study (OLAWIS) cohort compared to working adults [Canadian Community Health Survey (CCHS)], Ontario. *Italic Highlighting*: Pre-injury chronic condition prevalence statistically higher in OLAWIS cohorts than CCHS working adults. **Bold highlighting**: post-injury chronic prevalence statistically higher than pre-injury prevalence in OLAWIS cohorts.

	CCHS		OLAWIS1 ^a^								OLAWIS2 ^a^						
			Pre-Injury				Post-Injury				Pre-Injury				Post-Injury		
	Cases	Rate /100	Cases	Rate /100	95% CI		Cases	Rate /100	95% CI		Cases	Rate /100	95% CI		Cases	Rate /100	95% CI
Diabetes	653	4.8	94	6.1	4.8–7.7		108	7.0	5.6–8.7		59	6.2	4.6–8.3		71	7.4	5.6–9.6
Mood disorder	852	6.2	120	11.5	*9.4–13.9*		**216**	**19.4**	**16.7–22.4**		81	*12.9*	*10.1–16.2*		**131**	**20.7**	**17.2–24.8**
Asthma	1067	7.8	135	13.3	*11.1–15.9*		145	14.0	11.6–16.6		83	*12.6*	*9.9–15.8*		91	13.7	10.9–17.0
Migraine	1555	11.3	191	18.4	*15.7–21.4*		**296**	**28.5**	**25.2–32.3**		106	*15.9*	*12.9–19.4*		**188**	**28.7**	**24.6–33.4**
Hypertension	1622	11.8	168	10.8	9.1–12.8		**223**	**14.7**	**12.7–17.0**		107	11.4	9.2–14.1		**141**	**16.0**	**13.4–19.2**
Arthritis	1887	13.7	190	14.0	11.9–16.4		**336**	**24.8**	**22.0–28.0**		96	11.5	9.2–14.3		**163**	**19.1**	**16.0–22.6**
Back problems	2321	16.9	188	15.1	12.8–17.7		**379**	**32.3**	**28.8–36.0**		121	16.2	13.3–19.7		**225**	**31.7**	**27.4–36.4**

^a^ OLAWIS1 and OLAWIS2 prevalence rates age-standardized to CCHS age distribution.

For diabetes and asthma, age-standardized prevalence rates 18 months post-injury were equivalent to the pre-injury prevalence rates in both OLAWIS cohorts. However, the post-injury prevalence rates were substantially elevated for five conditions in both OLAWIS cohorts, representing the incidence of new physician diagnosis over the 18-month period. The prevalence rate for mood disorders increased from 11.5–12.9/100 pre-injury to 19.4–20.7/100 post-injury. The prevalence rate for migraine disorder increased from 15.9–18.4/100 pre-injury to 28.5–28.7/100, hypertension from 10.8–11.4/100 to 14.7–16.0/100, arthritis from 11.5–14.0/100 to 19.1–24.8/100 and back problems from 15.1–16.2/100 to 31.7–32.3/100.

Table 2 presents the distribution of demographic, employment, and health characteristics for incident chronic conditions post-injury. In the pooled cohorts, there were 18 incident cases of asthma, 213 incident cases of arthritis or back problems, 89 incident cases of hypertension or diabetes and 191 incident cases of migraine or mood disorder over the 18-month period post-injury. Although the distribution of incident asthma cases is reported in table 2, we have omitted further description here due to the small number of cases. Incident cases of arthritis/back problems and hypertension/diabetes were more common among older participants, and incident migraine/mood disorder more common among younger participants. In these three chronic condition categories, incident cases were more common among participants reporting high levels of pain interference with normal activities, among participants continuing to receive health care for treatment of the work-related injury and among participants who were not working at the 18-month interview post-injury. Additionally, incident cases in all three chronic condition categories were more likely to be members of the long benefit duration sample group.

**Table 2:** Distribution of independent variables, by incident chronic condition type, weighted sample. [M=mean; SD=standard deviation]

		Overall sample					Incident cases: asthma					Incident cases: arthritis or back problems					Incident cases: hypertension or diabetes					Incident cases: migraine or mood disorder			
		N	%	M	SD		N	%	M	SD		N	%	M	SD		N	%	M	SD		N	%	M	SD
Cases, weighted ^a^							18					178					77					155			
Cases, unweighted							18					213					89					191			
Sample, unweighted ^b^		1832					1605					1720					1753					1763			
Claimant age				46.4	13.2				52.1	12.7				52.7	10.8				50.9	11.2				42.2	12.4
Pain intensity																									
	None		28.4					16.1					9.0					13.9					12.6		
	A little bit to moderate		45.3					45.6					45.7					38.4					40.4		
	Quite a bit to extremely		25.8					38.3					45.0					47.8					46.7		
	Missing		0.5					0.0					0.3					0.0					0.3		
Age category (years)																									
	<40		33.3					16.1					14.6					16.4					47.3		
	40–49		19.6					8.9					13.9					14.5					17.1		
	50–59		30.1					57.8					44.4					46.3					28.1		
	≥60		17.0					17.3					27.1					22.8					7.5		
Sex																									
	Male		51.9					39.4					50.6					57.4					43.6		
	Female		47.9					60.6					49.4					42.6					56.4		
	Other/not specified		0.2					0.0					0.0					0.0					0.0		
Nature of injury																									
	Sprain, strain, or dislocation		45.5					41.4					46.0					36.7					36.1		
	Fracture		10.4					14.5					14.2					8.0					6.7		
	Superficial or open wound		14.1					29.9					12.6					17.6					7.4		
	Internal injury		13.5					0.0					10.2					15.7					31.0		
	Other		3.5					0.0					1.1					2.5					1.6		
	Missing		13.1					14.2					15.9					19.6					17.1		
Highest level of education																									
	Less than high school		24.4					3.4					23.3					19.1					27.1		
	High school completed		6.0					3.0					8.6					10.0					3.6		
	College or trade certificate/diploma		30.8					47.6					29.6					28.8					24.2		
	University degree		38.3					46.0					37.4					37.7					43.4		
	Missing		0.5					0.0					1.1					4.4					1.6		
Currently accessing healthcare for injury																									
	No		61.6					58.9					52.5					53.2					45.4		
	Yes		23.3					25.0					35.8					30.1					43.5		
	Not applicable		15.1					16.1					11.8					16.7					11.1		
Current employment type																									
	Permanent		69.8					78.4					64.9					77.7					64.3		
	Temporary (casual/contract/seasonal)		7.9					8.0					5.8					5.4					8.8		
	Not working		22.1					13.6					29.3					16.9					26.9		
	Missing		0.2					0.0					0.0					0.0					0.0		
Current work status																									
	Working, same job		52.7					62.3					47.8					54.0					42.9		
	Working, different job, same employer		7.7					16.1					10.4					17.8					9.7		
	Working, different employer		17.4					8.0					12.5					11.2					20.5		
	Not working		22.1					13.6					29.3					16.9					26.9		
Industry																									
	Healthcare and social assistance		16.5					21.9					13.4					15.8					18.6		
	Construction, utilities, mining, agriculture		12.6					16.1					12.5					11.6					13.0		
	Transportation & warehousing		14.1					25.6					16.3					13.6					11.8		
	Manufacturing		10.5					3.4					9.7					10.8					6.9		
	Other services (except public admin)		10.4					0.0					9.0					17.9					9.3		
	Retail, wholesale trade		8.6					16.1					7.9					9.5					9.7		
	Educational services		10.4					0.0					10.8					11.4					13.2		
	Accommodation/ food services/ arts		8.0					0.0					7.5					6.9					9.7		
	Public administration		6.1					17.0					8.8					2.5					5.0		
	Other		2.5					0.0					4.0					0.0					2.8		
OLAWIS2 cohort			43.8					49.3					39.3					44.1					50.3		
Benefit duration (>3 months)			35.4					27.7					42.2					37.6					44.3		

^a^ Proportions calculated in the weighted sample. Rounded proportions are presented. ^b^ Pre-injury cases removed from sample.

Table 3 reports the weighted, adjusted OR between persistent pain and the three incident chronic condition groups (arthritis and back problems, diabetes and hypertension, and migraine and mood disorders). The odds of reporting incident arthritis or back problems post-injury were higher among participants experiencing moderate levels (OR 3.82, 95% CI 1.83–7.97) or high levels (OR 7.71, 95% CI 3.47–17.13) of persistent pain. Similarly, the odds of reporting incident hypertension or diabetes post-work injury were elevated among participants experiencing high levels of persistent pain post-injury (OR 5.96, 95% CI 2.38–14.94) and the odds of reporting incident mood or migraine disorders post-injury were elevated among participants reporting moderate (OR 2.83, 95% CI 1.36–5.87) or high levels (OR 7.86, 95% CI 3.49–17.70) of persistent pain 18 months post-injury.

**Table 3:** Associations between persistent pain and incident chronic conditions, weighted logistic regressions, adjusted for all variables. **Bold values** indicate statistically significant estimates at the α = 0.05 level. [NA=insufficient sample size to estimate parameter. -- indicates collinear estimate].

		Arthritis & back problems (Yes vs. No) (N=1695/25^a^)				Hypertension and diabetes (Yes vs. No) (N=1728/25^a^)				Mood disorder & migraine (Yes vs. No) (N=1739/24^a^)		
		OR	95% CI	P-value		OR	95% CI	P-value		OR	95% CI	P-value
Intercept		**0.01**	**<0.01–0.03**	**<0.01**		**0.01**	**<0.01–0.04**	**<0.01**		**0.03**	**0.01–0.1**	**<0.01**
Age category (ref: <40 years)												
	40–49	1.43	0.67–3.07	0.35		1.19	0.40–3.52	0.75		**0.36**	**0.18–0.70**	**<0.01**
	50–59	**4.18**	**2.21–7.89**	**<0.01**		**4.03**	**1.65–9.84**	**<0.01**		**0.45**	**0.26–0.77**	**<0.01**
	≥60	**3.32**	**1.63–6.78**	**<0.01**		**3.03**	**1.07–8.62**	**0.04**		**0.14**	**0.05–0.38**	**<0.01**
Sex (female vs male)		1.19	0.72–1.97	0.50		0.88	0.47–1.66	0.70		1.36	0.80–2.30	0.26
Nature of injury (ref: sprain, strain, or dislocation)												
	Fracture	1.68	0.79–3.57	0.18		1.45	0.47–4.47	0.52		1.28	0.47–3.49	0.63
	Superficial or open wound	1.08	0.52–2.23	0.83		1.71	0.66–4.44	0.27		0.88	0.37–2.10	0.77
	Internal injury	1.16	0.58–2.29	0.68		**3.26**	**1.23–8.66**	**0.02**		**4.86**	**2.62–9.01**	**<0.01**
	Other	0.33	0.04–2.44	0.28		1.13	0.27–4.75	0.87		0.86	0.16–4.53	0.85
	Missing	1.40	0.74–2.65	0.30		**3.13**	**1.21–8.11**	**0.02**		1.96	0.97–3.98	0.06
Highest level of education (ref: university degree)												
	Less than high school	0.83	0.32–2.13	0.69		3.20	0.96–10.71	0.06		0.57	0.18–1.86	0.36
	High school completed	0.88	0.48–1.62	0.69		1.43	0.56–3.67	0.45		1.01	0.51–2.02	0.97
	College or trade certificate diploma	1.05	0.58–1.90	0.86		1.74	0.70–4.34	0.23		1.57	0.86–2.86	0.14
Pain level (ref: none)												
	A little bit to moderate	**3.82**	**1.83–7.97**	**<0.01**		1.59	0.62–4.04	0.33		**2.83**	**1.36–5.87**	**0.01**
	Quite a bit to extremely	**7.71**	**3.47–17.13**	**<0.01**		**5.96**	**2.38–14.94**	**<0.01**		**7.86**	**3.49–17.7**	**<0.01**
Currently receiving healthcare (ref: not applicable)												
	No	1.09	0.56–2.15	0.79		0.98	0.38–2.52	0.97		1.08	0.49–2.37	0.84
	Yes	1.55	0.72–3.30	0.26		0.86	0.32–2.35	0.77		1.76	0.79–3.93	0.17
Industry (ref: healthcare and social assistance)												
	Construction, utilities, mining, agriculture, forestry	1.14	0.45–2.93	0.78		0.50	0.15–1.69	0.27		1.12	0.45–2.76	0.81
	Transportation & warehousing	1.33	0.59–2.98	0.49		0.81	0.27–2.44	0.70		0.89	0.37–2.18	0.81
	Manufacturing	1.28	0.50–3.30	0.61		0.66	0.19–2.27	0.51		0.35	0.15–0.83	0.02
	Other services (except public admin)	0.89	0.32–2.46	0.82		0.97	0.34–2.75	0.96		0.40	0.15–1.06	0.07
	Retail, wholesale trade	0.99	0.38–2.59	0.98		0.68	0.18–2.51	0.56		1.02	0.37–2.80	0.97
	Educational services	0.95	0.39–2.31	0.91		0.82	0.23–2.90	0.76		0.92	0.41–2.05	0.84
	Accommodation/ food services/ arts/ entertainment	0.80	0.30–2.17	0.67		0.73	0.17–3.13	0.67		1.33	0.52–3.43	0.55
	Public administration	1.95	0.80–4.76	0.14		0.38	0.05–2.82	0.34		1.25	0.40–3.92	0.70
	Other	**3.04**	**1.02–9.07**	**0.05**		NA	NA	NA		0.82	0.25–2.76	0.75
	Not applicable (not currently working)	--	--	--		--	--	--		--	--	--
Employment status current (ref: permanent)												
	Temporary – casual contract seasonal	0.98	0.36–2.64	0.97		0.57	0.13–2.55	0.46		1.21	0.54–2.68	0.64
	Not working	1.18	0.70–2.00	0.53		0.40	0.16–1.04	0.06		1.11	0.61–2.01	0.73
	Cohort (2 vs 1)	0.83	0.53–1.28	0.40		0.84	0.43–1.65	0.62		1.38	0.87–2.20	0.17
	Benefit Duration (>3 months vs <3 months)	0.81	0.55–1.19	0.29		0.77	0.44–1.36	0.37		1.20	0.80–1.79	0.39
Prior chronic conditions (ref: none)												
	1	1.29	0.78–2.13	0.32		1.07	0.49–2.31	**0.87**		**0.51**	**0.29–0.90**	**0.02**
	2	1.23	0.70–2.18	0.47		0.68	0.27–1.72	0.41		0.57	0.28–1.14	0.11
	≥3	1.26	0.52–3.06	0.60		0.57	0.16–2.05	**0.39**		**0.28**	**0.10–0.80**	**0.02**

^a^ Number of observations deleted due to missing data.

Applying established approaches (40) for estimating the fraction of incident chronic conditions that may be attributed to persistent pain suggests that 37.0% of incident cases of arthritis or back problems, 38.2% of incident cases of hypertension or diabetes and 39.9% of incident cases of migraine or mood disorder may be attributed to exposure to high levels of persistent pain.

Supplementary table S1 (www.sjweh.fi/article/4096) reports unadjusted OR for the association of independent variables and incident chronic conditions, which were generally similar in magnitude to fully adjusted estimates.

## Discussion

In this sample of 1800 workers disabled by a work-related injury or illness, the prevalence of seven common chronic conditions pre-injury were either higher, or similar to prevalence estimates in a representative sample of working adults in the same setting. However, the incidence of five chronic conditions over the 18-month follow-up period was substantial in this cohort. After adjusting for demographic, health, and work-related factors, moderate and high levels of persistent pain at 18 months were associated with elevated chronic condition incidence.

The elevated incidence of five chronic conditions following a work-related injury or illness reported in this study is consistent with findings from other occupational cohort studies (6, 16, 17, 19). Empirical research in this field is not extensive, reflecting the challenges in implementing prospective occupational cohort studies that jointly ascertain the incidence of traumatic injury and the post-injury incidence of chronic conditions.

The prevalence of persistent pain among participants in this cohort was substantial, even after taking into account the oversampling of longer duration claims. The prevalence of severe persistent pain (pain reported in the last month) was roughly six times higher than the prevalence of high impact chronic pain (pain lasting ≥3 months) in adult populations in North America (23–25). Population attributable fractions were in the high 30% range for each chronic condition group studied. Severe persistent pain symptoms may be related to both causal hypotheses articulated in the introduction of this paper. Workers experiencing persistent pain symptoms will likely have more frequent health care encounters, which in turn may increase the probability of diagnostic investigation by health care providers and resulting diagnoses of previously unrecognized conditions. Alternatively, persistent pain may exacerbate or accelerate the onset of incident chronic conditions that are secondary to the original traumatic injury (41). For example, there is a well-established etiologic relationship between persistent pain and chronic mood disorders (42). There is also a substantial risk of the onset of post-traumatic osteoarthritis among workers experiencing a disabling work-related injury involving the musculoskeletal system (27).

Among workers disabled by a work-related injury, the utilization of healthcare services is frequently elevated beyond the period of acute management of the injury (43, 44). Given this pattern, enhanced clinician case-finding of undiagnosed chronic conditions is likely a component of the elevated 18-month incidence observed in these two cohorts. However, it may not plausibly account for the majority of the elevated incidence. For this to be true, the prevalence of undiagnosed chronic conditions in the OLAWIS cohorts of injured workers would have had to be substantial, implying deficits in the pre-injury health status of cohort members compared to all working adults in this setting. Among cohort members without pain symptoms at the 18-month interview, the proportion reporting poor or fair self-rated health (5.1%) was similar to the prevalence of poor/fair self-rated health among working adults in Ontario (6.0%, unpublished data, available from authors). While there is a limited number of longitudinal studies with health status measures obtained prior to traumatic injury, there is no consistent evidence that workers who experience a traumatic injury have substantial health status deficits prior to injury (5).

While pain interference prior to the work-related injury was not measured, it is unlikely that the high burden of persistent pain observed in this cohort of workers was prevalent prior to the injury. That all members of the cohort were actively employed at the time of the disabling injury or illness suggests a low prevalence of functional impairment prior to the injury. We note that respondents were specifically asked to describe pain intensity that they attributed to their disabling work-related injury, in addition to describing the degree of pain interference with normal activities.

There are several potential limitations that are relevant to guide the interpretation of the study’s findings. While we are confident that the study participants are demographically and occupationally representative of the population of injured workers’ compensation claimants in Ontario once sampling design probabilities have been accounted for, we cannot exclude the possibility that study participants had comparatively poorer health and functional status prior to the injury or at the time of the survey. If this was the case, estimates of the burden of persistent pain and the incidence of chronic conditions may not be representative of the population of injured workers. However, this potential sample selection bias does not limit the validity of the observed association between persistent pain and chronic condition incidence. Of more consequence, perhaps, are potential limitations in the reliability and validity of the measurement of persistent pain and the self-reported incidence of physician-diagnosed chronic conditions. Readers should recognize that the measure of persistent pain applied in this study of disabled workers may be vulnerable to over-estimation, with the consequence that the substantial associations between pain and the incidence of chronic conditions demonstrated in this study may have been overestimated. The estimated association between persistent pain and the incidence of chronic conditions has been adjusted for the nature of the disabling injury, anticipating that specific injury morbidity may be a potential confounder, as a cause of both persistent pain and the incidence of chronic conditions. However, the coarse classification of the nature of injury applied in our analysis may have resulted in residual confounding.

Related, it is possible that misclassification in chronic condition measures exist, in that each may be related to an underlying latent chronic condition. However, if this was the case, then separating this latent condition into separate disorders provides validity to our findings. There is some evidence that the probability of chronic condition incidence following traumatic injury is associated with injury severity (16). While this study did find a measure of disability episode duration was associated with the risk of chronic condition incidence, we were not able to implement a standardized measure of injury severity.

The ascertainment of incident chronic conditions, based on participant self-report of a physician diagnosis, may not be concordant with clinical records. The potential threat to the validity of the association between persistent pain and the incidence of chronic conditions is unknown. Further, the OLAWIS survey interview and context were different than that of the CCHS, potentially threatening the validity of comparison between the two samples. Finally, a portion of the post-injury incidence of chronic conditions described in this study may be transient, as several chronic conditions included in this study have a remitting and relapsing natural history (asthma, mood disorder, migraine, and back problems).

Among the strengths of this study is the inclusion of a reference population based on a representative sample of Ontario workers. The OLAWIS cohort design, interviewing workers 18 months following a disabling work-related injury, demonstrates the importance of longer duration prospective cohort studies in understanding the consequences of traumatic injury. We also note that the findings reported in this study have been replicated in two independent samples of injured workers.

### Concluding remarks

Policies regarding the benefit administration of work disability insurance schemes in Canada include provisions for entitlement for permanent impairment benefits for conditions that arise secondary to a disabling injury. These provisions, which align with the findings of this study, acknowledge the potential incidence of post-traumatic osteoarthritis, post-traumatic headache and psycho-traumatic disability following a disabling injury (45). The administrative process for applying for permanent impairment benefits relies on workers or their representatives to initiate an application for benefits, and the adjudication process can be arduous. In recent years, approximately 6% of workers who experience a disabling injury will apply for and receive permanent impairment benefits. While not all of the 500 incident cases of chronic disease identified in this cohort will have impairment consequences – nor would all incident cases have a plausible attribution as secondary to the disabling injury – the two cohorts included in this study have experienced an approximately 38% incidence rate for chronic conditions over an 18-month follow-up period. The findings of this study may have implications for the entitlement scope of work disability insurance schemes.

As noted above, empirical research on the association between occupational injury and the subsequent incidence of chronic conditions is limited. It appears this limitation is also present in the broader field of all-cause traumatic injury among adults of working age. The associations documented in this study are plausibly generalizable to all traumatic injuries experienced by working-aged adults (19) and suggest an important area for additional research attention. While the burden of all-cause traumatic injury is substantial in high-income countries (46), these burden estimates may underestimate the full consequences of traumatic injury on the health of adults of working age. Enhancements to national injury surveillance systems that incorporate longitudinal measures of health status in a post-injury follow-up period would strengthen estimates of the impact of traumatic injury on health and function.

## Supplementary material

Supplementary material
