# Supplementary material for: Association of persistent pain with the incidence of chronic conditions following a disabling work-related injury
Source: Scand J Work Environ Health. 2023 Jun 30;49(5):330–40. doi: 10.5271/sjweh.4096 (PMC10713986; doi:10.5271/sjweh.4096)
Supplement: Supplementary material [file SJWEH-49-330-S001.pdf]

Association of persistent pain with the incidence of chronic conditions following disabling work-related injury[1]

by Kathleen G Dobson, PhD,[2] Cameron A Mustard, ScD, Nancy Carnide, PhD, Andrea D Furlan, MD, PhD, Peter M Smith, PhD

1. *Supplementary table 1*
2. *Correspondence to: Kathleen G. Dobson, Institute for Work & Health, 400 University Ave, Suite 1800, Toronto, Ontario, Canada M5G 1S5, [E-mail: kdobson@iwh.on.ca]*

Crude Associations between persistent pain and incident chronic conditions, Weighted logistic regressions

	Arthritis & Back Problems			Hypertension and Diabetes			Mood Disorder & Migraine		
	(Yes vs. No)			(Yes vs. No)			(Yes vs. No)		
	OR	95% CI	p	OR	95% CI	p	OR	95% CI	p
**Pain Level (Ref: None)**									
A little bit to moderate	**4.19**	**2.06-8.52**	**<0.01**	1.47	0.58-3.72	0.41	**2.11**	**1.10-4.08**	**0.03**
Quite a bit to extremely	**9.70**	**4.73-19.89**	**<0.01**	**4.60**	**1.89-11.18**	**<0.01**	**5.19**	**2.71-9.95**	**<0.01**
**Age Category (ref: < 40)**									
40-49	1.89	0.9-3.98	0.09	1.50	0.51-4.46	0.46	**0.47**	**0.25-0.86**	**0.02**
50-59	**4.92**	**2.68-9.04**	**<0.01**	**4.52**	**2.00-10.24**	**<0.01**	**0.46**	**0.28-0.76**	**<0.01**
60 and over	**4.08**	**2.10-7.92**	**<0.01**	**3.47**	**1.31-9.20**	**0.01**	**0.19**	**0.08-0.44**	**<0.01**
**Sex (Female vs Male)**	1.19	0.79-1.80	0.40	0.96	0.52-1.76	0.89	1.52	0.99-2.34	0.06
**Nature of Injury (Ref: Sprain, Strain, or Dislocation)**									
Fracture	1.47	0.77-2.82	0.24	1.38	0.40-4.73	0.61	1.02	0.41-2.50	0.97

Superficial or Open Wound	1.02	0.53-1.94	0.96	1.60	0.66-3.90	0.30	0.73	0.29-1.82	0.50
Internal Injury	0.92	0.47-1.82	0.82	2.03	0.82-5.01	0.13	**3.82**	**2.23-6.52**	**<0.01**
Other	0.27	0.05-1.48	0.13	1.52	0.21-10.77	0.67	0.90	0.18-4.45	0.90
Missing	1.18	0.65-2.13	0.58	**2.59**	**1.12-5.99**	**0.03**	1.79	0.96-3.32	0.07
**Highest Level of Education (ref: University Degree)**									
Less than high school	1.41	0.58-3.43	0.45	**4.31**	**1.38-13.49**	**0.01**	0.38	0.14-1.04	0.06
High school completed	1.03	0.59-1.80	0.92	1.36	0.57-3.24	0.48	0.79	0.44-1.41	0.43
College or trade certificate diploma	1.12	0.66-1.92	0.67	1.43	0.62-3.30	0.40	1.17	0.70-1.95	0.55
**Currently Receiving Healthcare (ref: Not Applicable)**									
No	1.01	0.53-1.94	0.97	0.89	0.39-2.04	0.78	1.02	0.52-2.02	0.95
Yes	**2.35**	**1.18-4.69**	**0.02**	1.36	0.54-3.40	0.52	**2.85**	**1.42-5.74**	**<0.01**
**Industry (Ref: Health Care and Social Assistance)**									
Construction, Utilities, Mining, Agriculture, Forestry	1.03	0.46-2.29	0.95	0.62	0.21-1.83	0.39	0.98	0.45-2.12	0.95
Transportation & Warehousing	1.43	0.68-3.00	0.34	1.15	0.38-3.44	0.81	0.90	0.41-1.95	0.78
Manufacturing	1.33	0.56-3.16	0.52	1.11	0.34-3.65	0.86	**0.37**	0.15-0.88	**0.03**
Other Services (except Public Administration)	0.95	0.38-2.37	0.92	1.38	0.47-4.01	0.56	**0.37**	0.16-0.85	**0.02**

Retail, Wholesale Trade	0.99	0.4-2.42	0.98	0.91	0.27-3.12	0.89	1.32	0.56-3.08	0.52
Educational Services	1.32	0.58-3.00	0.51	1.09	0.35-3.43	0.88	1.06	0.51-2.18	0.88
Accommodation/ Food Services/ Arts/ Entertainment	0.91	0.34-2.42	0.85	1.05	0.28-4.01	0.94	1.24	0.52-2.97	0.62
Public Administration	1.84	0.76-4.42	0.18	0.49	0.07-3.67	0.49	0.93	0.33-2.63	0.89
Other	2.64	0.84-8.28	0.10	**<0.01**	**<0.01-<0.01**	**<0.01**	1.03	0.30-3.54	0.96
Not Applicable (Not Currently Working)	**<0.01**	**<0.01-<0.01**	**<0.01**	**<0.01**	**<0.01-<0.01**	**<0.01**	**<0.01**	**<0.01-<0.01**	**<0.01**
**Employment Status Current (Ref: Permanent)**									
Temporary - casual contract seasonal	0.88	0.37-2.10	0.77	0.54	0.14-2.12	0.38	1.22	0.57-2.63	0.61
Not working	**1.67**	1.05-2.66	**0.03**	0.66	0.28-1.55	0.34	1.32	0.81-2.16	0.26
**Cohort (2 vs 1)**	0.94	0.63-1.40	0.75	1.02	0.57-1.82	0.95	**1.59**	**1.04-2.42**	**0.03**
**Benefit Duration (> 3 Months vs < 3 Months)**	1.26	0.91-1.74	0.16	1.11	0.68-1.80	0.68	**1.63**	**1.15-2.31**	**0.01**
**Prior Chronic Conditions (Ref: None)**									
1 Condition	1.40	0.86-2.29	0.18	1.01	0.50-2.03	0.98	**0.57**	**0.34-0.98**	**0.04**
2 Conditions	1.67	0.95-2.94	0.08	0.89	0.38-2.09	0.79	0.62	0.33-1.15	0.13
3 + Conditions	2.03	0.96-4.33	0.07	0.87	0.24-3.13	0.83	0.42	0.15-1.16	0.10

Bold values indicate statistically significant estimates at the $\alpha = 0.05$ level
